# Pharmacological Activation of AMP-activated Protein Kinase Ameliorates Liver Fibrosis in a Metabolic Dysfunction-Associated Steatohepatitis Mouse Model

**DOI:** 10.7150/ijbs.108731

**Published:** 2025-04-21

**Authors:** Seojeong Kim, Jae-Ho Shin, Minjung Seo, Eun Seon Pak, Kyung-Hwa Jeon, Inhye Moon, Jisoo Kang, Wonhyo Seo, Younghwa Na, Youngjoo Kwon

**Affiliations:** 1 College of Pharmacy, Graduate School of Pharmaceutical Sciences, Ewha Womans University, Seoul, 03760, Republic of Korea.; 2 College of Pharmacy, CHA University, Pocheon, 487-010, Korea.; 3 Graduate Program in Innovative Biomaterials Convergence, Ewha Womans University, Seoul, 03760, Korea.

**Keywords:** AMP-activated protein kinase (AMPK), synthetic AMPK activator, 4-chloro-benzenesulfonamide derivative, metabolic dysfunction-associated steatohepatitis (MASH), liver fibrosis, lipid accumulation

## Abstract

Metabolic dysfunction-associated steatohepatitis (MASH) is a significant contributor to hepatocellular carcinoma (HCC). To validate AMPK activation as a therapeutic strategy for MASH-associated liver fibrosis, we investigated the effects of a 4-chloro-benzenesulfonamide derivative named KN21, a novel AMPK activator, on the liver fibrogenic process in a MASH model. In mice fed a choline-deficient, L-amino acid-defined, high fat diet (CDAHFD), KN21 reduced hepatic steatosis, lipid accumulation, and liver fibrosis. In hepatocyte cells treated with palmitic acid and oleic acid (PO), KN21 attenuated lipid accumulation and the release of reactive oxygen species (ROS) and fibrotic mediators. Hepatic stellate cells stimulated with hepatocyte-derived conditioned medium (CM) exhibited increased expression of fibrosis markers, whereas hepatic stellate cells exposed to CM from KN21-treated hepatocytes showed a decrease of fibrosis marker expression. Additionally, KN21 inhibited the activation of human hepatic stellate cells and demonstrated potent antifibrotic activity. These findings underscore the therapeutic potential of pharmacological AMPK activation for the treatment of MASH-associated liver fibrosis.

## Introduction

Metabolic dysfunction-associated steatotic liver disease (MASLD) is a chronic liver disease that affects over 30% of the worldwide population 1. MASLD encompasses a spectrum of disease states depending on the severity of liver damage, starting from simple steatosis (metabolic dysfunction-associated steatotic liver, MASL) and advancing to its inflammatory phase, known as metabolic dysfunction-associated steatohepatitis (MASH). Persistent MASH can further progress to cirrhosis and hepatocellular carcinoma 2. In MASH, the accumulation of free fatty acids (FFAs) in hepatocytes triggers the production of lipotoxic species, which in turn promotes the release of damage-associated molecular patterns (DAMPs), reactive oxygen species (ROS), and profibrotic mediators such as platelet-derived growth factor subunit B (PDGFB) and transforming growth factor-beta 1 (TGF-β1). This cascade activates hepatic stellate cells (HSCs), key drivers of liver fibrosis, through differentiation into myofibroblasts, resulting in abnormal deposition of extracellular matrix proteins, especially collagen 3-6. Recently, the U.S. Food and Drug Administration (FDA) approved the first drug, resmetirom, a liver-targeted thyroid hormone receptor-β selective agonist, for treating MASH patients with moderate to advanced liver fibrosis 7. However, there remains an urgent need for the development of alternative therapeutic approaches, as advanced liver fibrosis induced by MASH substantially raises the risk of liver disease-related complications and mortality 8.

AMP-activated protein kinase (AMPK) is a serine/threonine kinase structured as a heterotrimeric complex with a 1:1:1 ratio, comprising α, β, and γ subunits. Each subunit has multiple isoforms depending on the species and tissue types 9, 10. In humans, AMPK consists of two α- (α1, α2) and two β- (β1, β2) and three γ- (γ1, γ2, γ3) subunits, each encoded by distinct genes 11. Phosphorylation of the Thr172 residue within the activation loop of the N-terminal kinase domain of the AMPKα subunit is essential for full activation. Key upstream kinases responsible for AMPK phosphorylation at Thr172 include liver kinase B1 (LKB1), in response to a decreased ratio of ATP to AMP/ADP, and Ca^2+^/calmodulin-dependent protein kinase kinase-beta (CaMKKβ), in response to elevated Ca^2+^ levels 12-16. The generally accepted view regarding AMPK activation is that it is regulated by the binding of adenine nucleotides, including AMP, ADP, and ATP, based on structural analysis that identifies nucleotide binding sites within the γ-subunit of AMPK 9, 17, 18. The γ-subunit contains four cystathionine β-synthase (CBS) domains crucial for the binding of these nucleotides. The CBS-1, CBS-3, and CBS-4 domains are capable of binding with adenosine nucleotides, while the CBS-2 domain lacks this capability 9, 18-20. Under physiological conditions, AMP tightly binds to the CBS-4 domain in a non-exchangeable manner. The CBS-1 and CBS-3 domains serve as exchangeable binding sites, regulating AMPK activation through adenine nucleotide sensing. CBS-1 predominantly remains ATP-bound under physiological conditions. Furthermore, AMP binding to the CBS-3 domain, rather than CBS-1, induces a conformational change facilitating direct interaction with the α subunit 18, 19, 21. AMP binding to the γ-subunit activates AMPK through three mechanisms: it enhances LKB1-mediated phosphorylation at Thr172 of the activation loop, protects the activation loop from phosphatase-induced dephosphorylation, thereby sustaining AMPK activity, and triggers phosphorylation-independent allosteric kinase activation 19, 20, 22, 23. In the liver, the activation of AMPK is pivotal in governing lipid metabolism. On activation, AMPK inhibits the anabolic pathways involved in lipid synthesis, while simultaneously stimulating catabolic pathways, notably fatty acid oxidation (β-oxidation). This shift in metabolic balance leads to a reduction in lipid accumulation within hepatocytes in the liver. Overall, AMPK activation acts as a metabolic switch, promoting energy production and reducing lipid storage, which is beneficial for conditions associated with MASLD 24, 25.

Consequently, our research has focused on developing a novel direct AMPK activator that binds to the CBS-3 AMP binding site of the AMPKγ subunit, employing structural analysis-based computer-aided chemical design and chemical modification to enhance efficacy. Using this approach, we identified a 4-chloro-benzenesulfonamide derivative (named KN21) as a potent direct AMPK activator. We validated that pharmacological activation of AMPK by KN21 represents a promising strategy not only to alleviate steatosis but also to impede the progression of MASH. This therapeutic effect is achieved by diminishing lipid accumulation in hepatocytes and by inhibiting the activation, proliferation, and migration of HSCs, thereby reducing fibrosis in a choline-deficient, L-amino acid-defined high-fat diet (CDAHFD) mouse model.

## Materials and Methods

### KN21 synthesis

KN21 was synthesized and achieved a purity of 98.1%, confirmed by high-performance liquid chromatography (HPLC). Detailed information regarding the synthesis process along with the ^1^H and ^13^C nuclear magnetic resonance spectral data, HPLC chromatograms, and the liquid chromatography-high resolution mass spectroscopic data for KN21 can be found in the Supplementary Information (Scheme S1, Figs. S1-S3).

### CDAHFD-induced MASH mouse model

C57BL/6J mice (7 weeks old, male) were purchased from Raonbio (Gyonggi-do, South Korea). The control group received a normal chow diet, while the MASH model group was fed a high-fat diet consisting of L-amino acid rodent chow and 0.1% methionine (CDAHFD) for 6 weeks. KN21 (15 mg/kg) was administered intraperitoneally to the mice once every 2 days for a period of 4 weeks. All mice were anesthetized using 2,2,2-tribromoethanol (Sigma-Aldrich, MO, USA). After blood collection, the mice were euthanized by severing the inferior vena cava, allowing for perfusion of the entire body with PBS via the left ventricle. The liver was then harvested and weighed. The left lobe, comprising half of the largest lobe, was promptly fixed in 10% formalin. The remaining portions of the liver were rapidly frozen in liquid nitrogen. The animal studies were conducted in accordance with protocols approved by the Institutional Animal Care and Use Committee of Ewha Womans University (approval no.: 19-041).

### Cell culture and treatment

HepG2 cells were obtained from the ATCC (Manassas, VA, USA) and cultured in MEM (Welgene, Gyeongsangbuk-do, South Korea) supplemented with 10% FBS (Corning Inc., NY, USA) and 1% penicillin-streptomycin (HyClone, UT, USA) at 37°C in a 5% CO_2_ incubator. Primary hepatocytes were isolated from male C57BL/6J mice and cultured under similar conditions. For experimental treatment, both HepG2 cells and primary hepatocytes were exposed to a combination of 0.5 mM palmitic acid (#P0500, Sigma-Aldrich, MO, USA) and 1 mM oleic acid (#O1008, Sigma-Aldrich, MO, USA) in the presence of either KN21 or vehicle for a duration of 12 h prior to harvest. LX-2 cells were obtained from Sigma-Aldrich (MO, USA) and maintained in DMEM (Welgene, Gyeongsangbuk-do, South Korea) supplemented with 10% FBS and 1% penicillin-streptomycin at 37°C in a 5% CO_2_ incubator. Following a 12-h treatment period with TGF-β1 (R&D systems, MN, USA), LX-2 cells were further treated with KN21 and A769662 for an additional 12 h.

### Plasmid transfection

The AMPKγ1 gene was cloned into the p3XFLAG-Myc-CMV-26 plasmid. The AMPK H298G/R299F mutant was generated using a mutagenesis kit (Takara, Kyoto, Japan). Cells were transfected with these plasmids using jetPRIME® (Polyplus, Illkirch, France). The plasmids and transfection reagents were mixed in buffer and incubated at room temperature before application to cells.

### Production of hepatocyte-derived conditioned medium

Primary hepatocytes and HepG2 cells were treated with 0.5 mM palmitic acid and 1 mM oleic acid (PO) in the presence of either KN21 or vehicle for 12 h. Following treatment, the conditioned medium (CM) was collected and centrifuged at 1,500 rpm for 5 min. The resulting supernatant fractions were stored at -80°C until further use.

### Immunoblot analysis

Proteins from mouse liver and cells were immunolabeled with primary antibodies overnight at 4°C, followed by incubation with corresponding HRP-coupled secondary antibodies at room temperature. Images were obtained by a LAS-3000 (Fuji Photo Film Co., Ltd., Tokyo, Japan), equipped at Ewha Drug Development Research Core Center. The antibodies used in this study are listed in the Supplementary Information (Table S1).

### Cellular thermal shift assay

LX-2 cells were treated with DMSO or KN21 (5 μM) for 12 h. The cells were harvested, resuspended in PBS, and then divided into six PCR tubes. Subsequently, all samples in PCR tubes were heated to room temperature (RT), 45, 48, 50, 52 and 55°C for 3 min and then lysed using liquid nitrogen. The AMPKγ protein band was analyzed by immunoblotting. HEK293 cells were transfected with AMPK-WT and AMPK H298G/R299F mutant plasmids. After transfection, cells were treated with DMSO or KN21 (5 μM) for 12 h. The samples were heated to 48, 50, 51, 52, 53, 54 and 57°C for 3 min and then lysed using liquid nitrogen. Flag-tagged WT-AMPKγ and MU-AMPKγ proteins were subsequently detected through immunoblotting.

### *In silico* docking study

Ligand docking studies were performed using Cresset Flare^TM^ software V6.1 to confirm the direct binding potential and binding pose of KN21 to the AMPKγ subunit. The co-crystal structure of AMPK complexed with its ligand AMP was retrieved from the protein data bank (PDB code: 4RER). Prior to the docking process, KN21 was generated and subjected to energy minimization. For the docking study, the energy grid was defined based on the clustered endogenous AMP ligand at the CBS-3 site of AMPKγ1. The accuracy and reliability of the docking model were validated by evaluating the RMSD value of re-docked AMP in comparison to its original crystallographic orientation.

### Immunohistochemical analysis

For hematoxylin and eosin (H&E), the deparaffinized and rehydrated liver sections were sequentially stained with hematoxylin (#HMM999, ScyTek Inc., UT, USA) for 1 min and eosin (#318906, Sigma-Aldrich, MO, USA) for 1 min. For Sirius red staining, the deparaffinized and rehydrated liver sections were stained with picric acid (Abcam, Cambridge, UK) containing Fast-green FCF (Sigma-Aldrich, MO, USA) and 0.1% direct red 80 (Sigma-Aldrich, MO, USA) for 2 h. For immunohistochemistry analysis, the deparaffinized and rehydrated liver sections were incubated in antigen retrieval solution (#ab93678, Abcam, Cambridge, UK), followed by immersion in a 3% hydrogen peroxide solution and blocking serum buffer for 1 h. The primary antibodies were applied overnight at 4°C, followed by incubation with secondary antibodies for 30 min. The sections were developed using a Vectastain ABC kit (Vector Laboratories, CA, USA) for 30 min. Immunoreactions were visualized with DAB (Dako, Agilent, CA, USA) and the sections were counterstained with hematoxylin. Evaluation of the sections was performed using an Axiophot 2 apparatus (Carl Zeiss, Jena, Germany) at 200× magnification. Immunostaining was quantified using ImageJ software (NIH, MD, USA). The antibodies used in this study are listed in the Supplementary Information (Table S1).

### Determination of hepatic enzyme levels

The total concentrations of serum alanine aminotransferase (ALT) and aspartate aminotransferase (AST) were determined by analyzing serum samples using a chemistry analyzer (Hitachi 7020, Tokyo, Japan) in accordance with the manufacturer's instructions.

### Quantitative real-time polymerase chain reaction

Real-time quantitative polymerase chain reaction (qPCR) was performed using the SensiFAST^TM^ SYBR No-ROX kit (Bioline, London, UK) according to the manufacturer's protocol. Relative gene expression levels were determined using the *ΔΔ*Ct method and normalized to *GAPDH*. The primer sequences used in this study are summarized in the Supplementary Information (Table S2).

### Enzyme-linked immunosorbent assay (ELISA)

The levels of PDGFB (#DBB00, R&D Systems, Minneapolis, USA) and TGF-β1 (#DY240-05, R&D Systems, Minneapolis, USA) in the cell culture supernatants were quantified using ELISA kits, respectively, according to the manufacturer's instructions.

### Immunofluorescence staining

For immunofluorescence microscopy, slides were incubated overnight at 4°C with a primary antibody against SREBP-1c (Santa Cruz, CA, USA) and α-SMA (GeneTex, CA, USA). Then, the slides were treated with Alexa Fluor 488 (Abcam, Cambridge, UK) for 1 h, followed by staining with DAPI. Immunofluorescence images were obtained using a fluorescence microscope (Carl Zeiss, Jena, Germany), equipped at Ewha Drug Development Research Core Center.

### Statistical analysis

Statistical analyses were performed using GraphPad Prism statistical software (Version 6.01, GraphPad Software, Inc, CA, USA). All experiments were performed at least in triplicate to ensure reliability. In situations where more than one group was evaluated, ordinary one-way analysis of variance (ANOVA) followed by Tukey's post hoc test was applied. A *p*-value of less than 0.05 was considered statistically significant for all analyses.

The detailed methods and additional information for the other experiments are provided in the Supplementary Information.

## Results

### AMPK activator KN21 directly binds to the AMP binding site on the AMPKγ subunit

**Figure 1:**
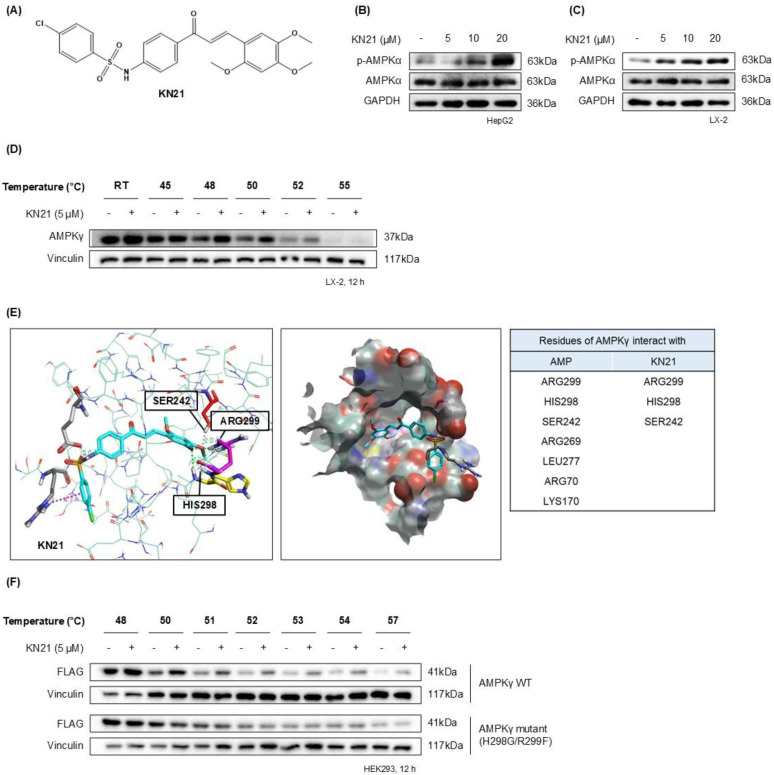
** KN21 stimulates AMPK activation by directly binding to the AMP binding site located on the AMPKγ subunit. (A)** Chemical structure of KN21, a 4-chloro-benzenesulfonamide derivative. **(B, C)** Concentration-dependent effects of KN21 on AMPKα phosphorylation in HepG2 and LX-2 cells, analyzed via immunoblotting. **(D)** Assessment of the interaction between KN21 and AMPKγ protein in LX-2 cells through cell thermal shift analysis (CETSA). **(E)** Docking studies to identify the interaction residues between AMPKγ and AMP as well as between AMPKγ and KN21. **(F)** CETSA assessment of the interaction between KN21 and both wild-type and mutant AMPKγ protein in HEK293 cells.

Considering the beneficial impact of AMPK activation in reducing hepatic lipid accumulation and mitigating metabolic conditions such as MASLD, we focused on developing a novel direct AMPK activator. Among the KN series compounds, KN21, a derivative of 4-chloro-benzenesulfonamide (Fig. 1A), was identified as the most potent AMPK activator. KN21 demonstrated greater potency than A769662, a known small molecule synthetic AMPK activator 9, 22, 26 used as a positive control, as determined by an *in vitro* AMPK kinase assay and immunoblotting analysis (data not shown). In both LX-2 HSCs and HepG2 hepatocyte cells, KN21 dose-dependently activated AMPK (Fig. 1B and C) without causing nonspecific toxicity at the treated concentrations (Fig. S4). To verify whether KN21 directly binds to the AMPKγ subunit, we employed a cellular thermal shift assay (CETSA), a well-established method that determines whether a compound binds directly to an endogenous protein in live cells by monitoring ligand binding-induced thermal stabilization 27. In LX-2 cells, KN21 treatment delayed the degradation of AMPKγ protein under heat stress, suggesting that KN21 stabilizes AMPKγ through direct binding (Fig. 1D). Further investigation using molecular docking revealed that KN21 binds specifically to the AMP binding site 3 (CBS-3) of the AMPKγ subunit, with key interactions involving SER242, HIS298, and ARG299 residues, identical to those facilitating AMP binding (Fig. 1E). Previous findings from our group demonstrated that HIS298 and ARG299 are essential for AMP binding-induced AMPK activation 28. In the cell line expressing mutant AMPKγ H298G/R299F, KN21 failed to stabilize AMPKγ protein (Fig. 1F). Collectively, the CETSA experiments in wild-type and mutant AMPKγ, combined with *in silico* molecular docking studies strongly support the notion that KN21 directly activates AMPK by binding to the CBS-3 domain of the AMPKγ subunit.

### KN21 mitigates hepatic steatosis, liver damage, and lipid accumulation in the CDAHFD-fed mouse model

**Figure 2:**
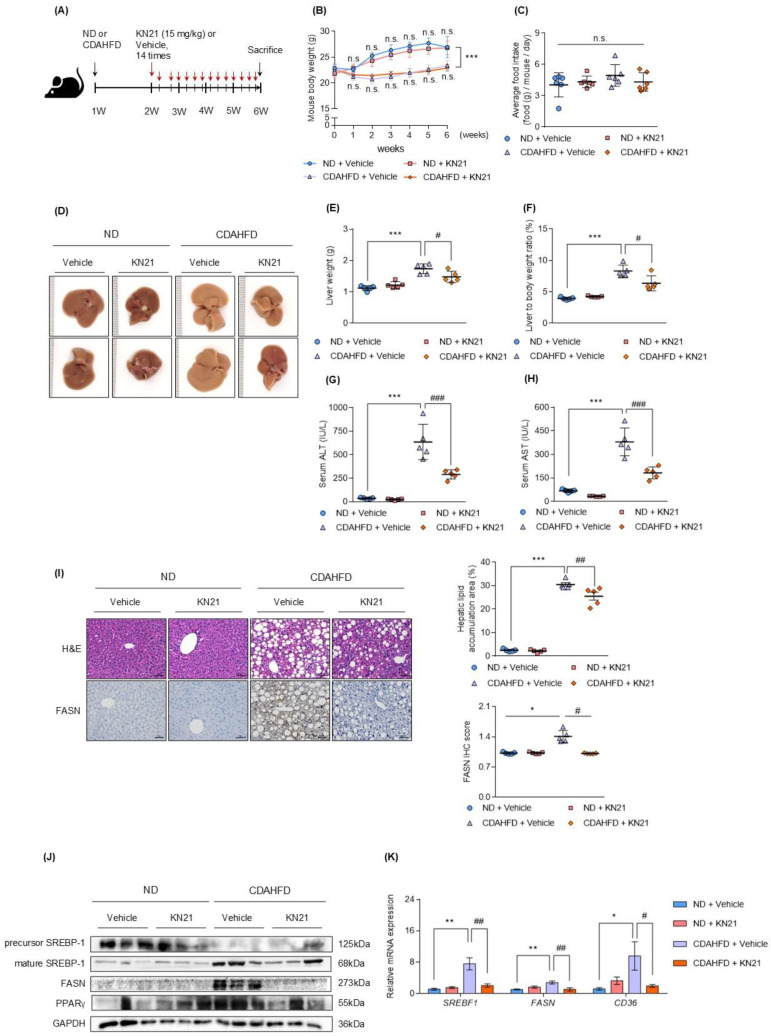
** KN21 mitigates hepatic steatosis, liver damage, and lipid accumulation in CDAHFD-fed mouse model. (A)** Experimental timeline showing CDAHFD feeding and KN21 (15 mg/kg) administration. **(B, C)** Body weight and food intake of mice across treatment groups during the experimental period (n=5). **(D)** Representative liver images from each group (n=5). **(E, F)** Liver weights and liver-to-body weight ratio for each group (n=5). **(G, H)** Serum levels of ALT and AST, indicators of liver function (n=5). **(I)** Representative images of H&E and immunohistochemical staining for FASN in liver sections (n=5). **(J)** Immunoblotting analysis of proteins involved in lipid metabolism (SREBP-1, FASN and PPARγ) (n=5, combined with results in Figure S6A). **(K)** Quantitative real-time PCR analysis of lipid metabolism-related genes, including *SREBF1, FASN*, and *CD36* (n=5). ***P < 0.001, **P < 0.01, *P < 0.05 vs. the ND group; ^###^*P* < 0.001, ^##^*P* < 0.01, ^#^*P* < 0.05 vs. the CDAHFD group (one-way ANOVA); “n.s.” indicates a nonsignificant difference.

To investigate the therapeutic effects of KN21 on MASH *in vivo*, mice were fed a CDAHFD for 2 weeks, followed by KN21 administration while continuing CDAHFD feeding for an additional 4 weeks. Mice fed a normal chow diet (ND) were used as controls (Fig. 2A). KN21 treatment did not significantly affect body weight (Fig. 2B) or food intake (Fig. 2C). However, it notably reduced liver weight (Fig. 2D, E, and S5) and the liver-to-body weight ratio (Fig. 2F) and significantly decreased serum ALT and AST levels (Fig. 2G and H), indicating reduced liver damage. Considering that AMPK activation plays a critical role in mitigating hepatic lipid accumulation 7, 24, we investigated whether KN21 attenuated hepatic lipid deposition as an AMPK activator. Histological analysis by H&E demonstrated that KN21 treatment alleviated hepatic steatosis in CDAHFD-fed compared to the vehicle-treated group (Fig. 2I). Furthermore, KN21 reduced the elevated levels of fatty acid synthase (FASN), a key enzyme involved in de novo fatty acid synthesis 29. Proteins associated with lipid metabolism and lipogenesis, including sterol regulatory element binding transcription factor 1c (SREBP-1c), FASN and peroxisome proliferator-activated receptor gamma (PPARγ) 30, were significantly downregulated following KN21 treatment (Fig. 2J and S6A). Similarly, KN21 markedly reduced the expression of lipogenesis and lipid metabolism-related genes, such as *SREBF1*, *FASN*, and cluster of differentiation 36 (*CD36*) 31, which were elevated in CDAHFD-fed mice (Fig. 2K). Collectively, these findings suggest that KN21 effectively reduces hepatic lipid deposition in CDAHFD-fed mice, likely through AMPK-mediated pathways.

### The reduction in hepatic steatosis and liver fibrosis induced by KN21 in the CDAHFD mouse model is attributed to AMPK activation

**Figure 3:**
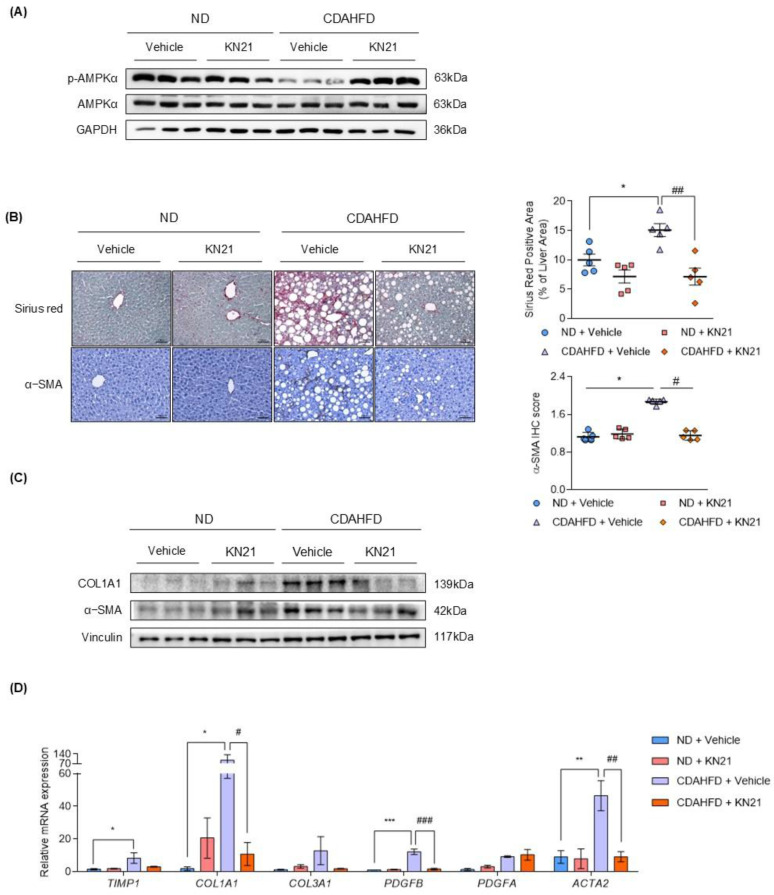
** KN21 improves liver fibrosis induced by CDAHFD in mice as an activator of AMPK. (A)** Immunoblotting analysis showing total and phosphorylated AMPKα proteins in CDAHFD-fed mice treated with 15 mg/kg of KN21 (n=5, combined with results in Figure S6B). **(B)** Representative images of Sirius red staining and immunohistochemical staining for α-SMA in liver sections (n=5). **(C)** Immunoblotting analysis of proteins involved in fibrosis (COL1A1 and α-SMA) (n=5, combined with results in Figure S6C). **(D)** Quantitative real-time PCR analysis of fibrosis-related genes (*TIMP1*, *COL1A1*, *COL3A1*, *PDFGB, PDGFA, ACTA2*) (n=5). ***P < 0.001, *P < 0.05 vs. the ND group; ^###^*P* < 0.001, ^##^*P* < 0.01, ^#^*P* < 0.05 vs. the CDAHFD group (one-way ANOVA).

We examined whether KN21 inhibits liver fibrosis through AMPK activation. In CDAHFD-fed mice, p-AMPK levels were reduced but this reduction was reversed by KN21 treatment, as confirmed by the immunoblotting (Fig. 3A and S6B) and immunohistochemistry analyses (Fig. S7). Increased liver fibrosis in CDAHFD-fed mice, as evidenced by Sirius red staining, was significantly alleviated by KN21 treatment. Immunohistochemistry analysis further confirmed that elevated α-SMA levels in the livers of CDAHFD-fed mice decreased upon KN21 treatment (Fig. 3B). KN21 lowered the protein expression of fibrotic markers, including collagen type 1 alpha 1 chain (COL1A1) and α-SMA (Fig. 3C and S6C), which were otherwise heightened in CDAHFD-fed mice. Real-Time PCR analysis demonstrated that the mRNA expression of fibrosis markers, including *COL1A1*, *PDGFB,* and *ACTA2,* was significantly downregulated in KN21-treated mice. Meanwhile, *TIMP1* and *COL3A1* exhibited a downward trend, although statistically insignificant, and *PDGFA* expression remained unaffected (Fig. 3D). Collectively, these results indicate that KN21 effectively mitigates liver damage and fibrosis induced by lipid accumulation in CDAHFD-fed mice. To compare the *in vivo* therapeutic potency of KN21 and A769662, we administered intraperitoneal injections of KN21 (15 mg/kg) and A769662 (30 mg/kg) for two weeks in a CDAHFD-induced MASH mouse model (Fig. S8A), based on prior evidence that KN21 demonstrated approximately 2-fold higher AMPK-activating efficacy than A769662 in cell-based assays. Neither KN21 nor A769662 significantly affected body weight or food intake during the treatment period (Fig. S8B and C). However, both compounds significantly reduced liver weight and the liver-to-body weight ratio to a similar extent (Fig. S8D-F), and markedly decreased serum ALT and AST levels, indicating improved liver function (Fig. S8G and H). Notably, total cholesterol, glucose, insulin levels, white adipose tissue weight, and the adipose-to-body weight ratio did not differ significantly between CDAHFD-fed and ND-fed mice (Fig. S8I-M). These data suggest that while KN21 and A769662 effectively reduced liver weight, their impact on overall adipose tissue mass remained minimal in this model. Importantly, KN21 significantly increased hepatic p-AMPK levels in CDAHFD-fed mice, to a degree comparable to A769662, despite being administered at half the dose (Fig. S8N). Collectively, these results indicate that KN21 exerts hepatoprotective effects comparable to A769662 *in vivo*, through AMPK activation.

### AMPK activation by KN21 reduces lipid accumulation in hepatocytes under metabolic stress

**Figure 4:**
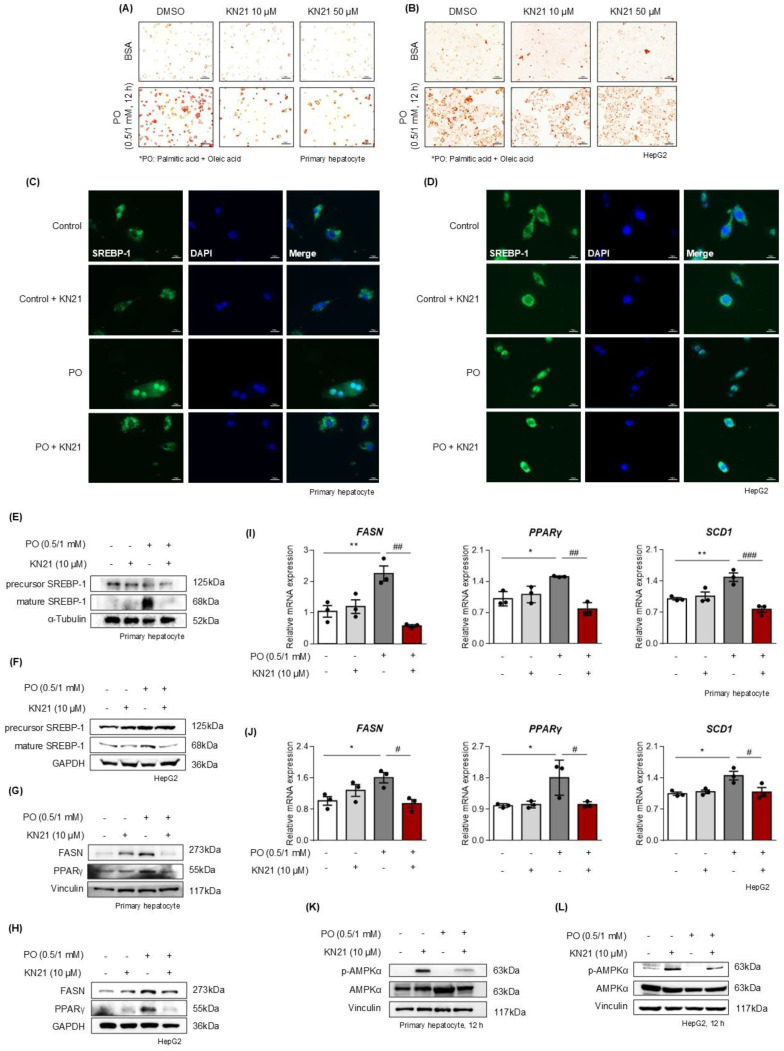
** KN21 inhibits lipid accumulation in PO-induced hepatocytes. (A, B)** Oil red O staining in primary hepatocytes and HepG2 cells stimulated with BSA or PO (0.5 mM PA and 1.0 mM OA), and subsequently treated with DMSO (vehicle), 10 μM KN21, or 50 μM KN21 for 12 h. **(C, D)** Immunofluorescence staining of SREBP-1 in PO-stimulated primary hepatocytes and HepG2 cells treated with 10 μM KN21 for 12 h.** (E, F)** Immunoblotting analysis of precursor and mature forms of SREBP-1 in PO-stimulated primary hepatocytes and HepG2 cells treated with 10 μM KN21 for 12 h. **(G, H)** Immunoblotting analysis of FASN and PPARγ in primary hepatocytes and HepG2 cells stimulated with PO and treated with 10 μM KN21 for 12 h. **(I, J)** Quantitative real-time PCR analysis of lipid metabolism-related genes (*FASN*, *PPARγ*, and *SCD1*) in PO-stimulated primary hepatocytes and HepG2 cells treated with 10 μM KN21 for 12 h. **(K, L)** Immunoblotting analysis showing total and phosphorylated AMPKα proteins in PO-stimulated primary hepatocytes and HepG2 cells treated with 10 μM KN21 for 12 h. **P < 0.01, *P < 0.05 vs. the control group; ^###^*P* < 0.001, ^##^*P* < 0.01, ^#^*P* < 0.05 vs. the PO-stimulated group (one-way ANOVA)..

Considering that hepatocytes are primarily responsible for managing lipotoxicity induced by fatty acid 32, we examined the direct effects of KN21 on hepatocytes under metabolic stress conditions. Primary hepatocytes and HepG2 cells were exposed to palmitic acid and oleic acid (PO) stimulation, with or without KN21 treatment. Oil red O staining revealed a significant increase in cellular lipid droplet accumulation in PO-treated primary hepatocytes and HepG2 cells compared to BSA-treated control cells, which was markedly reduced by KN21 treatment (Fig. 4A and B). The lipid-lowering effect of KN21 was confirmed by measuring intracellular TG levels (Fig. S9A). In primary hepatocytes and HepG2 cells stimulated with PO, KN21 treatment reduced both the nuclear translocation of SREBP-1c and the protein level of its nuclear active form (68 kD), which were elevated under lipotoxic conditions (Fig. 4C-F). These effects were consistent with the reduced expression of FASN and PPARγ by KN21 (Fig. 4G and H). Furthermore, KN21 downregulated fatty acid synthesis-related genes, including *FASN*, *PPARγ*, and stearoyl-CoA desaturase-1 (*SCD1*) (Fig. 4I and J). As expected, KN21 treatment increased the p-AMPK levels in both primary hepatocytes and HepG2 cells (Fig. 4K and L), confirming its ability to activate AMPK signaling under PO-induced metabolic stress.

### Inhibition of lipid accumulation by KN21 in hepatocytes under metabolic stress is mediated through AMPK activation

**Figure 5:**
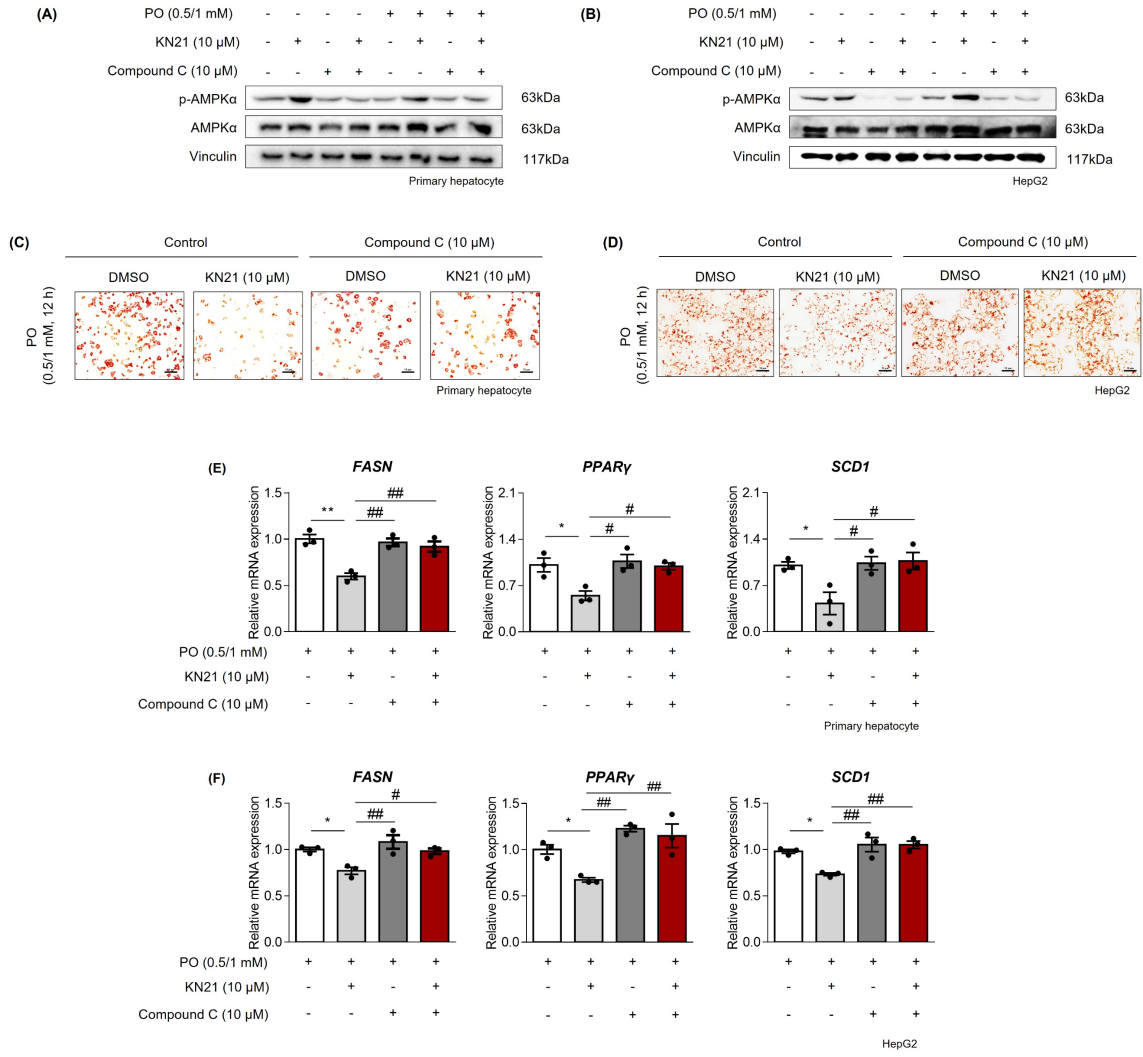
** KN21 reduces lipid accumulation in hepatocytes under metabolic stress via AMPK activation. (A, B)** Immunoblotting analysis showing total and phosphorylated AMPKα proteins in PO-stimulated primary hepatocytes and HepG2 cells treated with KN21, Compound C, or their combination for 12 h. **(C, D)** Oil red O staining of PO-stimulated primary hepatocytes and HepG2 cells treated with KN21, Compound C, or their combination for 12 h, indicating lipid accumulation. **(E, F)** Quantitative real-time PCR analysis of lipid metabolism-related gene expression (*FASN*, *PPARγ*, and *SCD1*) in PO-stimulated primary hepatocytes and HepG2 cells treated with KN21, Compound C, or their combination for 12 h. **P < 0.01, *P < 0.05 vs. the PO-stimulated group; ^##^*P* < 0.01, ^#^*P* < 0.05 vs. the PO + KN21-treated group (one-way ANOVA).

To confirm that the lipid-lowering effects of KN21 are mediated through AMPK activation, hepatocytes were co-treated with KN21 and Compound C, an AMPK inhibitor. Compound C significantly inhibited KN21-induced AMPK phosphorylation in PO-stimulated hepatocytes (Fig. 5A and B). Oil red O staining revealed that while KN21 effectively reduced lipid accumulation in PO-treated primary hepatocytes and HepG2 cells, this effect was reversed in the presence of Compound C (Fig. 5C and D). Furthermore, KN21 treatment significantly reduced intracellular TG levels; however, this lipid-lowering effect was abolished in the presence of Compound C (Fig. S9B). Similarly, Compound C abrogated the KN21-induced downregulation of fatty acid synthesis-related genes, including FASN, PPARγ, and SCD1 (Fig. 5E and F). These findings confirm that the lipid-lowering effects of KN21 in hepatocytes under metabolic stress are dependent on AMPK activation.

### KN21 inhibits ROS production and the secretion of profibrotic mediators from damaged hepatocytes

**Figure 6:**
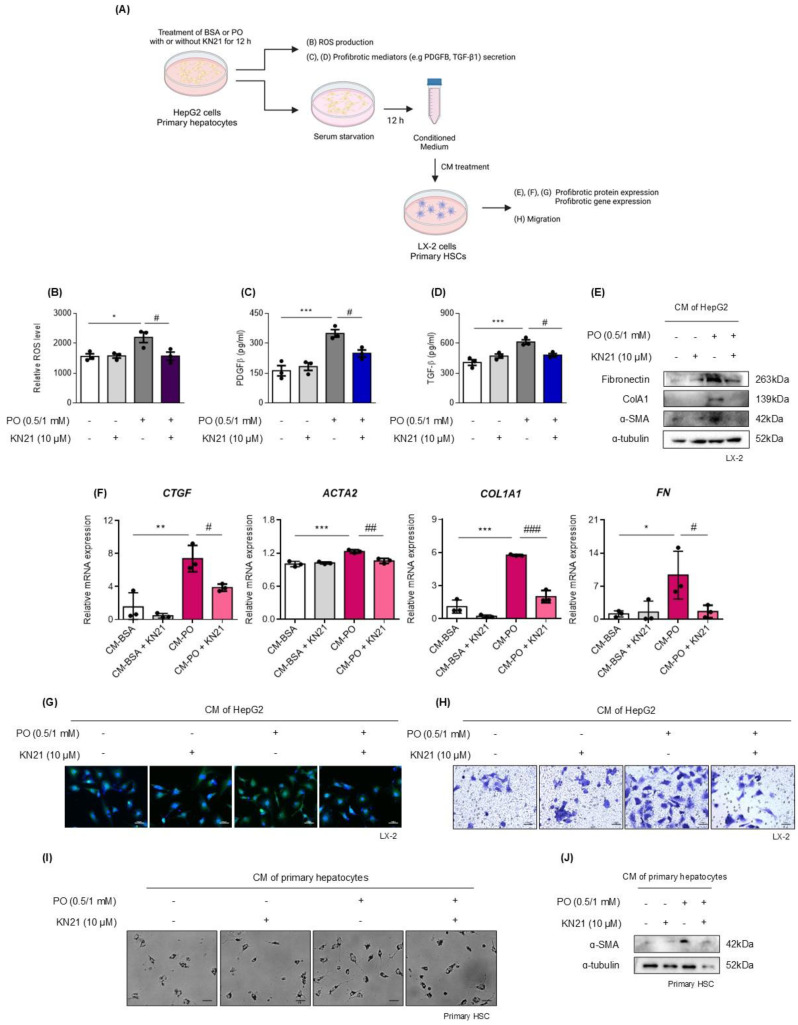
** KN21 suppresses ROS production and the release of profibrotic mediators from hepatocytes. (A)** Schematic representation measuring ROS production and the release of profibrotic mediators in hepatocytes. **(B)** Measurement of intracellular ROS production in HepG2 cells. **(C, D)** Quantification of the profibrotic mediators PDGFβ and TGF-β1 in the culture media from HepG2 cells. **(E)** Immunoblotting analysis of fibrosis-related proteins (Fibronectin, COL1A1 and α-SMA) in LX-2 cells treated with CM derived from HepG2 cells. **(F)** Quantitative real-time PCR analysis of profibrotic gene expression (*CTGF*, *ACTA2*, *COL1A1*, *FN*) in LX-2 cells treated with CM derived from HepG2 cells. **(G)** Immunofluorescence staining of LX-2 cells treated with CM derived from HepG2 cells, showing α-SMA (green) in confocal images. **(H)** Transwell migration assay of LX-2 cells treated with CM derived from HepG2 cells. **(I)** Morphological images of primary HSCs treated with CM derived from primary hepatocytes. **(J)** Immunoblotting analysis of α-SMA protein expression in primary HSCs treated with CM derived from primary hepatocytes. ***P < 0.001, **P < 0.01, *P < 0.05 vs. the control group; ^###^*P* < 0.001, ^##^*P* < 0.01, ^#^*P* < 0.05 vs. the PO-treated group (one-way ANOVA).

We assessed whether damaged hepatocytes promote ROS production and the release of profibrotic mediators (Fig. 6A). PO treatment significantly increased intracellular ROS levels, which were subsequently reduced by KN21 (Fig. 6B). ELISA analysis of the cell supernatant revealed that PO treatment increased the secretion of profibrotic mediators, PDGFB and TGF-β1, and this effect was attenuated by KN21 (Fig. 6C and D). Additionally, LX-2 cells treated with CM derived from PO-treated hepatocytes exhibited increased levels of profibrotic proteins, including fibronectin (FN), COL1A1, and α-SMA, which were markedly reduced when exposed to CM derived from KN21-treated hepatocytes (Fig. 6E). The expression of profibrotic genes in LX-2 cells was similarly reduced upon treatment with KN21-treated hepatocytes-derived CM (Fig. 6F). Immunofluorescence staining and trans-well migration assays further confirmed that KN21-treated hepatocyte-derived CM decreased the number of α-SMA positive cells and inhibited LX-2 cell proliferation and activation (Fig. 6G and H). The primary HSCs treated with CM derived from PO-treated primary hepatocytes were activated during culture, characterized by distinct stellate morphology and increased proliferation. This proliferative response was markedly reduced upon exposure to CM derived from KN21-treated primary hepatocytes (Fig. 6I). Consistently, the expression of α-SMA in primary HSCs was similarly reduced upon treatment with CM from KN21-treated primary hepatocytes (Fig. 6J). These findings highlight the potential of KN21 to mitigate ROS production and suppress the release of profibrotic mediators from hepatocytes, thereby limiting the activation and proliferation of HSCs.

### KN21 effectively reduces the activation of hepatic stellate cells

**Figure 7:**
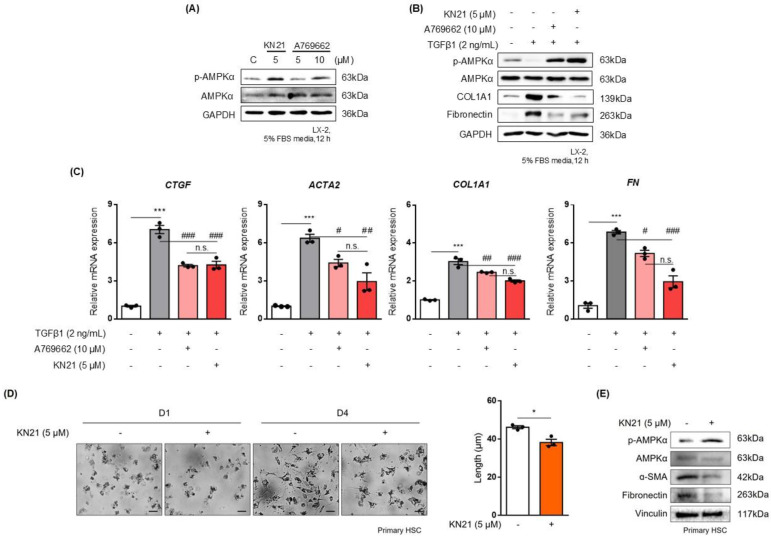
** KN21 reduces TGF-β1-induced activation of hepatic stellate cells. (A)** Immunoblotting analysis showing total and phosphorylated AMPKα proteins in LX-2 cells treated with 10 μM A769662 or 5 μM KN21 for 12 h. **(B)** Immunoblotting analysis of protein expression in LX-2 cells treated with 2 ng/mL TGF-β1 for 12 h, followed by incubation with 10 μM A769662 or 5 μM KN21 for 12 h. **(C)** Quantitative real-time PCR analysis of fibrosis-related gene expression (*CTGF*. *ACTA2*, *COL1A1*, *FN*) in LX-2 cells treated with 2 ng/mL TGF-β1 for 12 h, followed by incubation with 10 μM A769662 or 5 μM KN21 for 12 h. (D) Morphological images of primary HSCs cultured for a specific period. **(E)** Immunoblotting analysis of protein expression in primary HSCs treated with 5 μM KN21. ***P < 0.001 vs. the control group; ^###^*P* < 0.001, ^##^*P* < 0.01, ^#^*P* < 0.05 vs. the TGF-β1-treated group (one-way ANOVA).

To mimic the effects of hepatocyte-derived CM, LX-2 cells were treated with TGF-β1. Treatment with 5 μM KN21 induced p-AMPK levels comparable to those achieved with 10 μM A769662, a well-known direct AMPK activator (Fig. 7A). KN21 significantly attenuated the increased levels of profibrotic markers, COL1A1 and FN, induced by TGF-β1, demonstrating a more potent antifibrotic activity compared to A769662 (Fig. 7B). In addition, KN21 reduced the expression of profibrotic genes, including *CTGF*, *ACTA2*, *COL1A1*, and *FN* (Fig. 7C). To evaluate the inhibitory effect of KN21 on HSC activation, primary HSCs were isolated from C57BL/6J mice. Freshly isolated primary HSCs initially exhibited a round morphology with visible lipid droplets, characteristic of quiescent cells. Over time in culture, these cells gradually transitioned to a stellate morphology, indicative of activation. This morphological change was accompanied by increased expression of fibrotic markers, including α-SMA and FN, confirming HSC activation. Treatment with KN21 attenuated these changes, as evidenced by reduced expression of α-SMA and FN (Fig. 7D and E). These findings underscore the efficacy of KN21 in inhibiting HSC activation, emphasizing its potential as a therapeutic agent for liver fibrosis.

## Discussion

The contemporary lifestyle, characterized by excessive caloric intake, has contributed to the rising prevalence of MASLD 33. MASLD, closely associated with obesity, is marked by the abnormal buildup of fat in the liver, posing a significant risk factor for various metabolic disorders 34. As MASLD progresses from simple steatosis (MASL) to MASH, it displays histological features such as steatosis, ballooning, inflammation, and fibrogenesis, which are key markers that define the pathophysiology of MASH 35. The progression from MASH to liver fibrosis is particularly concerning as it significantly elevates the risk of mortality related to liver complications 8. Therefore, identifying therapeutic targets to prevent the progression of MASH to liver fibrosis is of critical importance.

AMPK activity is generally well maintained in healthy livers and normal hepatocytes 36. Unlike isolated hepatocytes, normal liver tissue consists of a heterogeneous population of cells, including hepatocytes, Kupffer cells, and hepatic stellate cells, which may contribute to variable AMPK activation responses. In contrast, isolated hepatocytes provide a more controlled environment where changes in p-AMPK can be more readily detectable. Decreased AMPK activity has been reported in MASLD 36 and similarly, the CDAHFD-fed mice in our study demonstrated decreased AMPK activity. In the CDAHFD-fed mice, where hepatic AMPK activity is significantly suppressed, KN21 treatment led to a pronounced increase in p-AMPK levels, indicating its ability to restore AMPK activation in a metabolically compromised liver. These findings suggest that KN21 exerts a more pronounced effect on AMPK activation in damaged liver conditions, such as the CDAHFD-induced liver injury, compared to healthy liver tissue, where AMPK activity is already maintained at functional levels. Moreover, Genetic mouse models with liver-specific AMPK activation have shown that AMPK activation can inhibit MASLD progression 37, highlighting AMPK as a promising therapeutic target for MASH. Various AMPK activators, including A769662, pioglitazone, and PF-06409577, have been demonstrated to reduce hepatic steatosis 37-39. Furthermore, PXL770, a specific AMPK activator, has advanced to phase II clinical trials, where it was observed to decrease *de novo* lipogenesis, liver lipids, blood glucose, and insulin resistance in individuals with type 2 diabetes 40. These findings underscore the critical role of AMPK activation in inhibiting hepatic lipid accumulation and reducing fibrosis.

AMPK is structured as a heterotrimer, comprising a catalytic α-subunit and two regulatory subunits (β and γ). The binding of AMP to the AMPKγ subunit leads to allosteric activation, enhancing AMPK activity up to 10-fold under physiological ATP levels 41. This binding also shields the phosphorylation at Thr172, essential for full AMPK activation from dephosphorylation. Our research revealed that KN21, a novel AMPK activator, directly interacts with the γ-subunit to induce AMPK activation. KN21 increased p-AMPK levels and enhanced AMPK activity in a dose-dependent manner, as confirmed by the *in vitro* AMPK kinase assay. In the CDAHFD-induced MASH mouse model, KN21 significantly increased AMPK phosphorylation and effectively improved both hepatic steatosis and fibrosis. Notably, these improvements were observed at doses lower than those required for A769662, a well-known direct AMPK activator 36. However, KN21 did not significantly ameliorate inflammation in our model. Despite this, the reduction in hepatic lipid accumulation by KN21 distinctly alleviated liver fibrosis, aligning with the effects observed with other AMPK activators 36, 39. This underscores the critical role of AMPK activation in reducing lipid accumulation. Furthermore, AMPK activation has been reported to prevent the proteolytic processing of SREBP, thereby inhibiting the expression of key enzymes in fatty acid and triglyceride synthesis, such as FASN and SCD1 42.

Excessive FFAs are central to MASH pathogenesis. When adipose tissue's capacity to store fat becomes compromised, it leads to elevated levels of circulating FFAs 43. These FFAs, originating from adipose tissue, are transported through the bloodstream to the liver 44. Lipids in the liver are primarily stored as triglycerides, an inert and non-cytotoxic lipid form. However, the buildup of toxic intermediates, including saturated FFAs, their derivatives, and complex lipids like lysophosphatidylcholine and ceramides, contributes to lipotoxicity 45. The buildup of these lipids disrupts cellular function through various mechanisms, including oxidative stress, impaired mitochondrial function, and initiation of apoptosis. As non-adipose tissues have limited capacity for FFA storage, the enzymes necessary for β-oxidation can become depleted 46. Therefore, efficient processing mechanisms, such as esterification to triglycerides and enhanced β-oxidation, are critical for managing excess FFAs.

Among the sterol regulatory element-binding protein (SREBP) family, SREBP-1a and SREBP-1c are crucial transcription factors responsible for regulating the expression of genes involved in fatty acids synthesis. SREBP-2, on the other hand, activates genes related to cholesterol metabolism. SREBP-1a is primarily expressed in specific immune cell types, while SREBP-1c is mainly expressed in the liver 47. SREBP is produced as a precursor protein and remains inactive while associated with the SREBP cleavage activating protein (SCAP) and insulin inducer gene-1 (Insig-1) within the endoplasmic reticulum (ER) 48. When cellular cholesterol levels decrease, the SREBP-SCAP complex dissociates from Insig-1 and translocates to the Golgi apparatus. Proteases then cleave SREBP, causing it to translocate into the nucleus and promote the expression of lipogenic genes. Our current research demonstrates that under metabolic stress conditions, KN21 exhibited remarkable ability to inhibit lipid accumulation by suppressing SREBP. In contrast, the AMPK inhibitor, compound C, abolished this protective effect of KN21, confirming that KN21's lipid-lowering effects are mediated through AMPK activation.

Hepatocytes, which account for up to 80% of the total hepatic cell population, are intricately connected with both arterial and venous blood 49, 50. Therefore, the pathophysiology of liver diseases must consider interactions between different types of hepatic cells. Excess accumulation of lipids and cholesterol in hepatocytes leads to the production of various fibrotic mediators, as well as free radicals like ROS. The secretion of these fibrotic mediators and ROS affects the activation of HSCs 3, 51. The activation of HSCs is also crucial in advancing fibrosis 52. Quiescent HSCs, upon activation, differentiate into myofibroblasts, acquiring traits such as proliferation, contraction, migration, and fibrogenesis 53. During this transition, activated HSCs lose their lipid droplets and concurrently exhibit a significant increase in the synthesis of extracellular matrix components, including collagen types I, III, and IV, fibronectin, α-SMA. Activated HSCs can be eliminated through apoptosis, senescence, or reversion to an inactive phenotype 54. Therefore, we proceeded to investigate whether damaged hepatocytes could affect the activation of HSCs. Our data showed that the expression of genes associated with HSC activation was notably increased in LX-2 cells exposed to medium derived from PO-treated hepatocytes. We confirmed that KN21 mitigated metabolic stress in hepatocytes, thereby reducing ROS production and the release of fibrotic mediators. Furthermore, KN21 decreased the expression of fibrosis markers in HSCs exposed to CM derived from hepatocytes.

TGF-β1 is the most well-known fibrotic mediator that causes the initial activation of HSCs 55. In our study, KN21 demonstrated the ability to inhibit TGF-β1-induced HSC activation. KN21 not only activated AMPK but also exerted anti-fibrotic effects on HSCs at lower concentrations compared to A769662, highlighting AMPK activation as a mechanism to attenuate HSC activation and fibrosis 56, 57. These findings provide substantial evidence of the beneficial role of KN21 in both hepatocytes and HSCs. By activating AMPK, KN21 inhibited lipid accumulation in hepatocytes, decreased ROS generation and fibrotic mediator release, and consequently suppressed HSC activation. Thus, KN21 holds potential for alleviating liver fibrosis.

In conclusion, our findings suggest that KN21 exerts multifaceted benefits in MASH by targeting AMPK activation. By reducing lipid accumulation, ROS production, and HSC activation, KN21 addresses key aspects of MASH pathogenesis. These results highlight the therapeutic promise of KN21 and reinforce the value of AMPK activators in treating MASH-associated liver fibrosis.

## Supplementary Material

Supplementary methods, figures and tables.

## Abbreviations

ND: normal chow diet
CDAHFD: a choline-deficient, L-amino acid-defined, high fat diet
TIMP1: TIMP metallopeptidase inhibitor 1
α-SMA: α-smooth muscle
COL1A1: collagen type 1 alpha 1 chain
COL3A1: collagen type III alpha 1 chain
PDGFB: platelet-derived growth factor subunit B
PDGFA: platelet-derived growth factor subunit A
ACTA2: actin alpha 2
FASN: fatty acid synthase
SREBP-1c: sterol regulatory element binding protein-1c
TGF-β1: transforming growth factor beta 1
PPARγ: peroxisome proliferator-activated receptor gamma
SCD1: stearoyl-CoA desaturase-1
CTGF: connective tissue growth factor
FN: fibronectin
AMPK: AMP-activated protein kinase
