# Supplementary material for: Pharmacological Activation of AMP-activated Protein Kinase Ameliorates Liver Fibrosis in a Metabolic Dysfunction-Associated Steatohepatitis Mouse Model
Source: Int J Biol Sci. 2025 Apr 21;21(7):2957–72. doi: 10.7150/ijbs.108731 (PMC12080390; doi:10.7150/ijbs.108731)
Supplement: Supplementary file 1 — Supplementary methods, figures and tables. [file ijbsv21p2957s1.pdf]

Supplementary information

Pharmacological Activation of AMP-activated Protein Kinase Ameliorates Liver Fibrosis in a Metabolic Dysfunction-Associated Steatohepatitis Mouse Model

Seojeong Kim,[1,#] Jae-Ho shin,[2] Minjung Seo,[1] Eun Seon Pak, [1,3] Kyung-Hwa Jeon, [1,3] Inhye Moon,[1,3] Jisoo Kang,[1] Wonhyo Seo,[1, 3] Younghwa Na,[2,*] and Youngjoo Kwon,[1,*]

Contents

Synthesis of KN21

Scheme S1. Synthesis of **KN21**

Supplementary Materials and Methods

Figure S1. ^{1}H-NMR Spectrum of **KN21**

Figure S2. ^{13}C-NMR Spectrum of **KN21**

Figure S3. HPLC chromatogram of **KN21**

Figure S4. Cytotoxicity of **KN21** and A769662 in HepG2 cells.

Figure S5. Additional liver images to complete the representation of all experimental groups in Figure 2 (n = 5).

Figure S6. KN21 reduces lipogenesis (A) through AMPK activation (B) and mitigates liver fibrosis (C) in CDAHFD-fed mouse model.

Figure S7. Immunohistochemical staining of phosphorylated AMPK (p-AMPKα) in liver sections (n=5).

Figure S8. KN21 and A769662 mitigate hepatic steatosis and liver damage in CDAHFD-fed mice via AMPK activation.

Figure S9. Effects of KN21 on lipid accumulation in primary hepatocytes.

Table S1. Antibodies used in this study

Table S2. Primer Sequences used in this study

Synthesis of KN21

Chemicals and reagents used were obtained from Aldrich Chemical Co. and TCI. Chromatographic separations were monitored by thin-layer chromatography using a commercially available pre-coated Merck Kieselgel 60 F254 plate (0.25 mm) and detected by visualizing under UV at 254 and 365 nm. Silica gel column chromatography was carried out with Merck Kieselgel 60 (0.040-0.063 mm). All solvents used for chromatography were directly used without distillation. The purity was assessed by HPLC (Shimadzu LC-20AD) analysis under the following conditions; column, SunFire C18 (4.6 mm × 150 mm, 5 mm); mobile phase, A (water) and B (acetonitrile) using an isocratic condition of 60% B in 0-15 min, flow rate; 1.0 mL/min; detection, diode array detector (Shimadzu Spd-M20A). The purity of compound is described as percent (%) and retention time was given in minutes. NMR spectra were recorded on AVANCE NEO Nanobay NMR Spectrometer (^{1}H NMR at 400 MHz and ^{13}C NMR at 100 MHz) with tetramethylsilane as an internal standard. Chemical shift (δ) values are expressed in ppm and coupling constant (*J*) values in hertz (Hz). Melting points were measured without correction in open capillaries with Barnstead Electrothermal melting point apparatus, Manual MELTEMP (Model No: 1202D). Mass spectral investigations were performed on a Agilent 6230 TOF LC/MS (Agilent Technology, Santa Clara, CA, USA) equipped with an electrospray ionization (ESI) source.

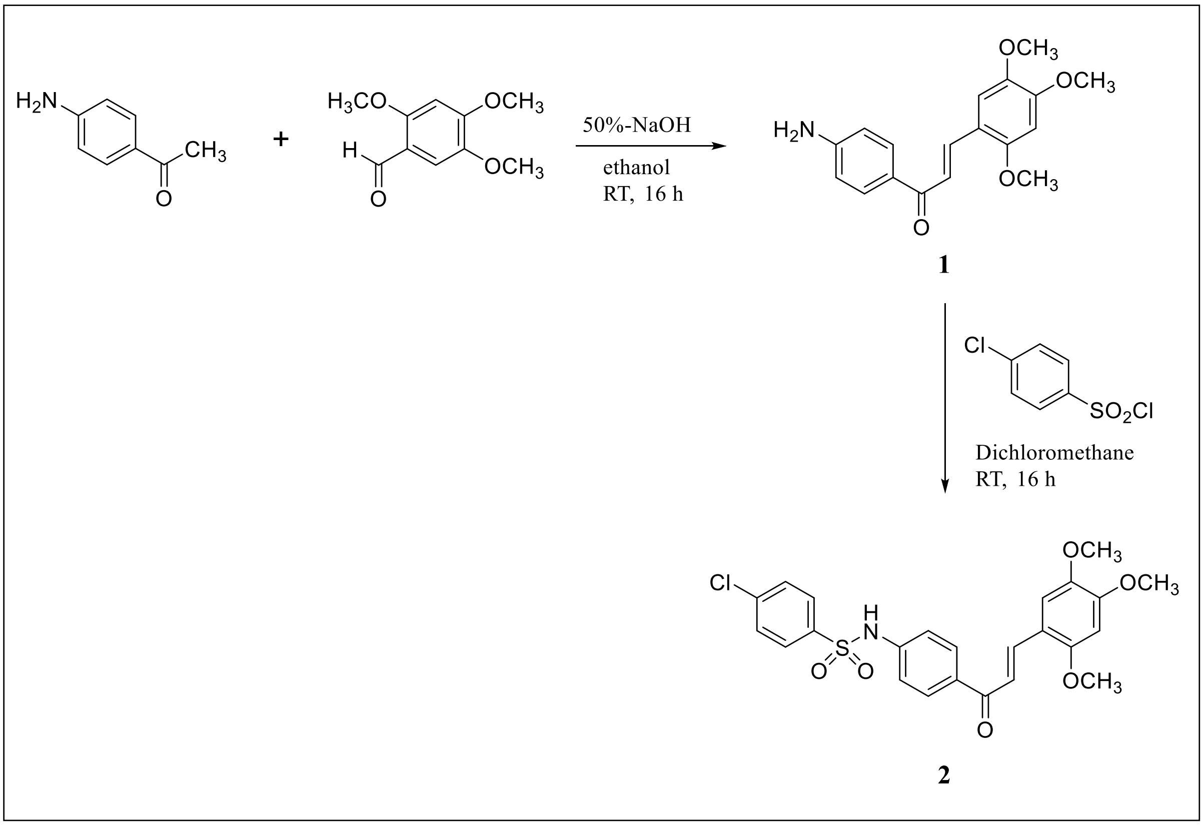

Scheme S1. Synthesis of **KN21 (2)**

(*E*)-1-(4-Aminophenyl)-3-(2,4,5-trimethoxyphenyl)prop-2-en-1-one (1)

The reaction mixture of 4-amino acetophenone (1.00 g, 7.40 mmol), 2,4,5-trimethoxy benzaldehyde (1.45 g, 7.40 mmol), and 50% NaOH (2.96 mL 36.99 mmol) in EtOH (50 mL) was stirred at room temperature (16 h) and water was added. The reaction mixture was extracted with ethyl acetate and organic layer was collected and washed with brine, and then dried over anhydrous $MgSO_4$. Solvent was removed under reduced pressure and the residue was purified by silica gel chromatography (ethyl acetate : dichloromethane : *n*-hexane = 1:1:1) to give compound **1** (1.37 g, 59.1%) as a yellow solid. m.p. 194-198 °C; R_f 0.29 (ethyl acetate : dichloromethane : *n*-hexane = 1:1:1); HPLC: R_T 2.69 min (purity: 98.4%); ^{1}H-NMR(DMSO, 400MHz) δ 3.82 (s, 3H), 3.86 (s, 3H), 3.88 (s, 3H), 6.07 (s, 2H), 6.62 (d, *J* = 8.4 Hz, 2H), 6.73 (s, 1H), 7.47 (s, 1H), 7.69 (d, *J* = 15.6 Hz, 1H), 7.91 (d, *J* = 8.4 Hz, 2H), 7.94 (d, *J* = 15.6 Hz, 1H); ^{13}C-NMR(DMSO, 100MHz) 55.8, 56.3, 56.4, 97.7, 110.9, 11.7, 114.8, 119.2, 125.8,

130.9, 135.9, 143.1, 152.1, 153.5, 153.6, 186.1 ppm.

(*E*)-4-Chloro-*N*-(4-(3-(2,4,5-trimethoxyphenyl)acryloyl)phenyl)benzenesulfonamide (2)

To a mixture of compound 1 and 4-chlorobenzene sulfonyl chloride (1.01 g, 4.79 mmol) were added dry dichloromethane (50 mL) and triethylamine (0.53 mL, 3.83 mmol) under N_2 atmosphere. The reaction mixture was stirred at room temperature (16 h) and extracted with dichloromethane and sat. $NaHCO_3$. Organic layer was collected and washed with water, and then dried over anhydrous $MgSO_4$. Solvent was removed under reduced pressure and the residue was purified by silica gel chromatography ((ethyl acetate : dichloromethane : *n*-hexane = 1:1:1)) to give compound **2** (380 mg, 24.4%) as a yellow solid. m.p. 232~236 °C; R_f 0.45 (ethyl acetate : dichloromethane : *n*-hexane = 1:1:1); HPLC: R_T 8.56 min (purity: 98.1%); ^{1}H-NMR(DMSO, 400MHz) δ 3.80 (s, 3H), 3.86 (s, 3H), 3.88 (s, 3H), 6.73 (s, 1H), 7.25 (d, *J* = 8.8 Hz, 2H), 7.47 (s, 1H), 7.67 (d, *J* = 8.8 Hz, 2H), 7.68 (d, *J* = 15.6 Hz, 1H), 7.83 (d, *J* = 8.8 Hz, 2H), 7.99 (d, *J* = 15.6 Hz, 1H), 8.03 (d, *J* = 8.8 Hz, 2H), 10.94 (s, 1H); ^{13}C-NMR(DMSO, 100MHz) 55.8, 56.3, 56.4, 97.5, 110.9, 114.3, 118.3, 118.4, 128.6, 129.6, 130.0, 133.4, 138.0, 138.1, 138.2, 141.6, 143.1, 152.9, 154.2, 187.4 ppm; HRMS-ESI (m/z) $[M+H]^+$ C24H23ClNO6S calcd 488.0929, found 488.0928.

Supplementary Materials and Methods

Cell viability assay

Cells were seeded in 96-well plates and incubated with 100 μL of medium. The following day, cells were treated with various concentrations of the compounds for 24 h. After treatment, 5 μL of EZ-CytoX (DoGen, Seoul, Korea) was added to each well and incubated for approximately 4 h. The absorbance was measured at 450 nm using the Infinite M200 PRO Microplate Reader (Tecan Group Ltd., Männedorf, Switzerland). IC_{50} values were calculated using Table Curve 2D program (SPSS, Chicago, USA).

AMPK kinase assay

AMPK activity was measured using the AMPK kinase assay kit (Promega, Madison, USA) and the ADP-Glo^{TM} assay kit (Promega, Madison, USA). According to the manufacturer's protocol, the AMPK enzyme, SAMS peptide, and ATP were mixed in kinase buffer and incubated at RT for 1 h to initiate the AMPK kinetic reaction, which phosphorylates SAMS by converting ATP to ADP. The ADP-Glo^{TM} reagent was added to each well and incubated for 40 min to deplete any remaining unreacted ATP. The kinase detection reagent was then added to each well and incubated for 30 min to convert ADP to ATP. Finally, the light intensity generated from the newly synthesized ATP, using a luciferase/luciferin reaction, was measured with the Infinite M200 PRO Microplate Reader (Tecan Group Ltd., Männedorf, Switzerland), equipped at the Ewha Drug Development Research Core Center.

Cellular ROS assay

ROS levels were assessed using a DCFDA/H2DCFDA-cellular ROS assay kit (#ab113851,

Abcam, Cambridge, UK) as per the manufacturer's instructions. Cells were seeded in each well of a 96-well plate. After treatment with PO alone or in combination with 10 μM KN21, the media was aspirated, and cells were incubated with 5 μ M DCFDA diluted in clear media for 30 min at 37 °C. ROS levels were measured using the Infinite M200 PRO Microplate Reader (Tecan Group Ltd., Männedorf, Switzerland) at 485 nm excitation and 530 nm emission wavelengths.

Transwell migration assay

LX-2 cells, treated with HepG2-derived CM, were seeded in the upper chambers of a transwell plate (8 mm pore size, #3428, Corning, NY, USA), while the bottom chambers were filled with 5% FBS media. After a 24 h of incubation, the migrated LX-2 cells were fixed with 4% paraformaldehyde and stained with a crystal violet solution (1 % [w/v] in absolute methanol).

Cellular oil red O staining

Cells were washed 2-3 times with PBS and fixed with 4% formaldehyde at RT for 10 min. Then, the cells were stained with a 60% oil red O solution (O1391, diluted with water; Sigma-Aldrich) at RT for 5 min.

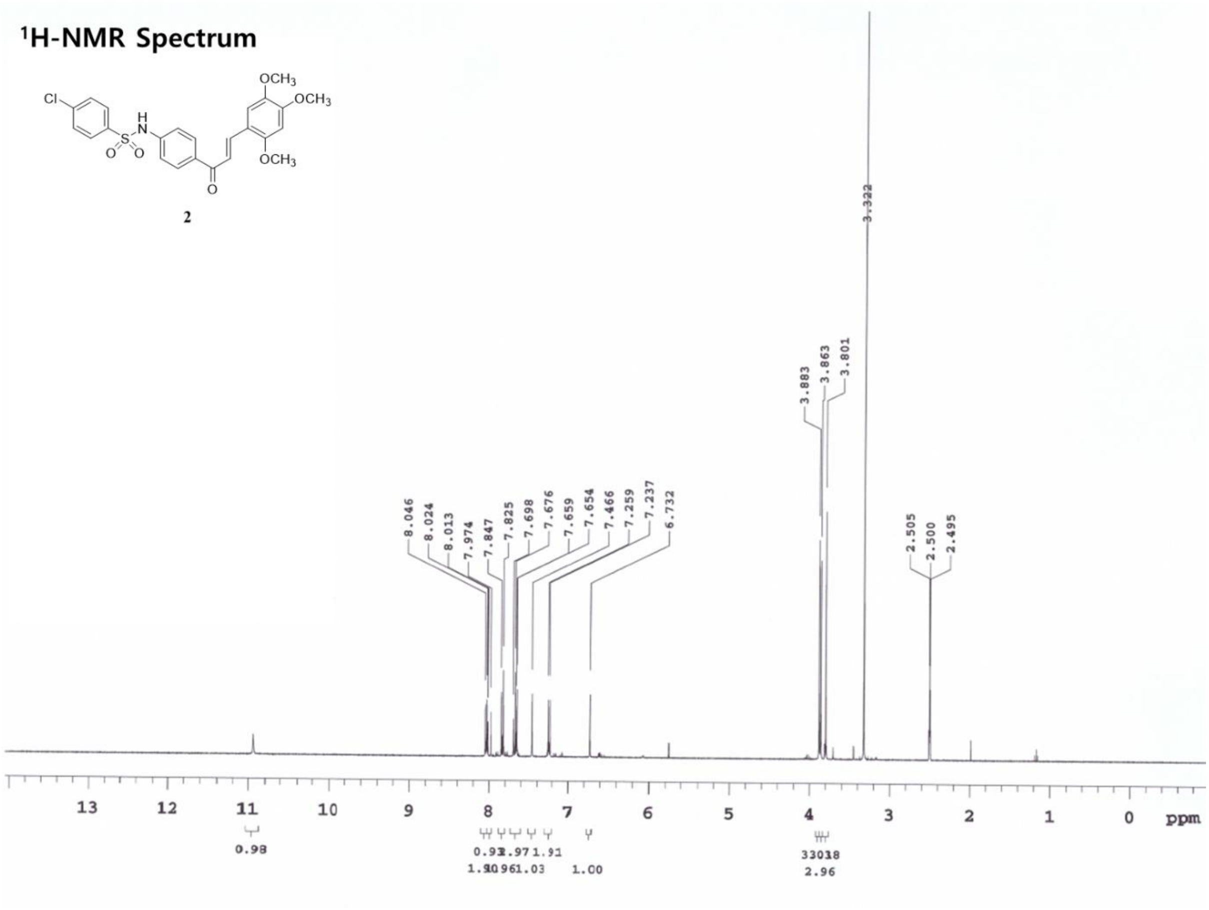

Figure S1. ¹H-NMR Spectrum of **KN21**

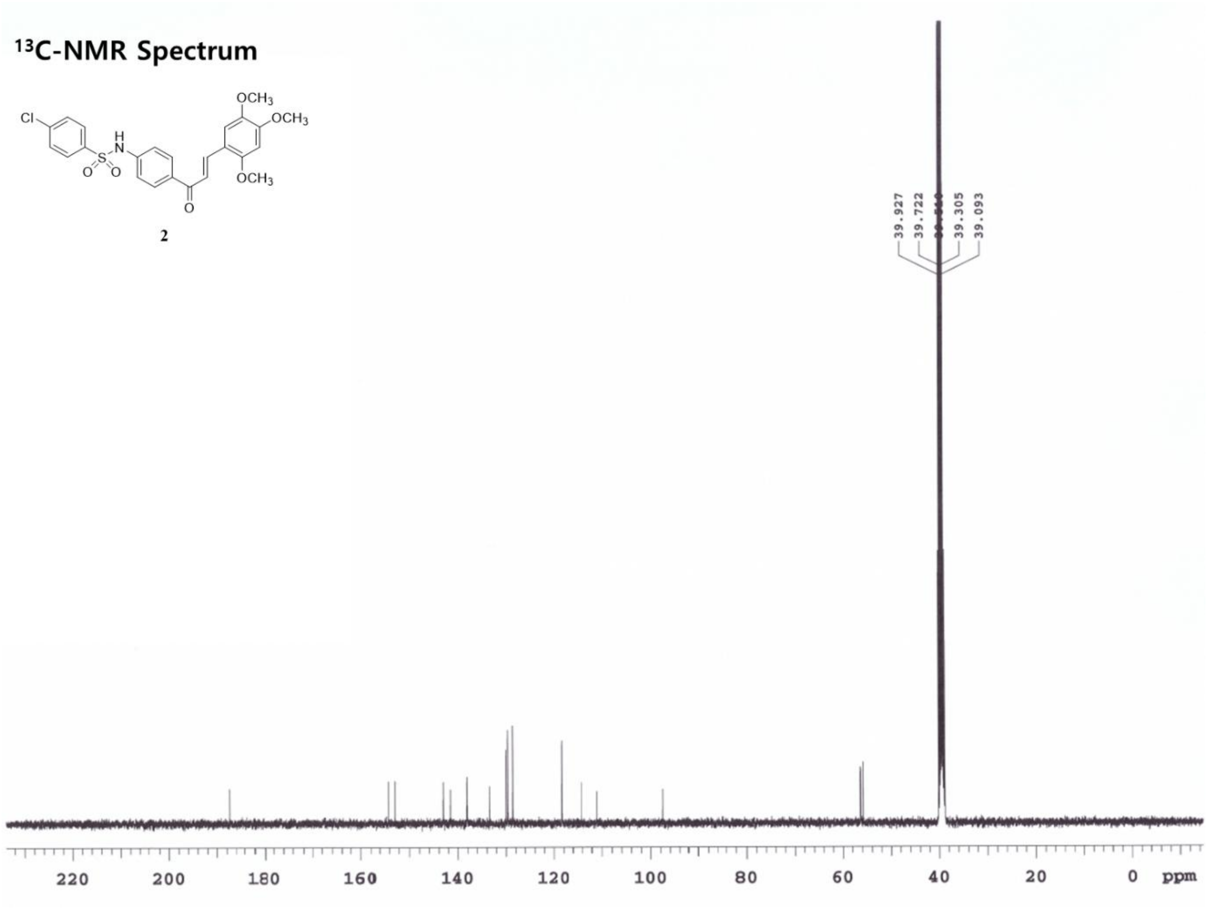

Figure S2. ^{13}C-NMR Spectrum of **KN21**

<Chromatogram>

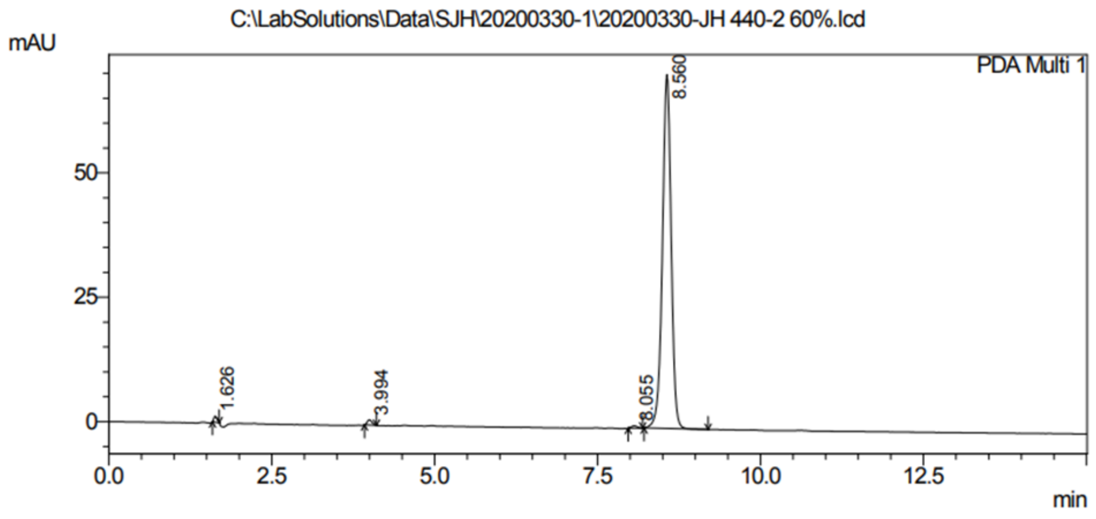

Peak#	Ret. Time	Area	Height	Area %	Height %
1	1.626	4544	1179	0.672	1.599
2	3.994	5166	1030	0.764	1.397
3	8.055	2878	440	0.426	0.597
4	8.560	663098	71083	98.137	96.407
Total		675685	73732	100.000	100.000

Figure S3. HPLC chromatogram of **KN21**

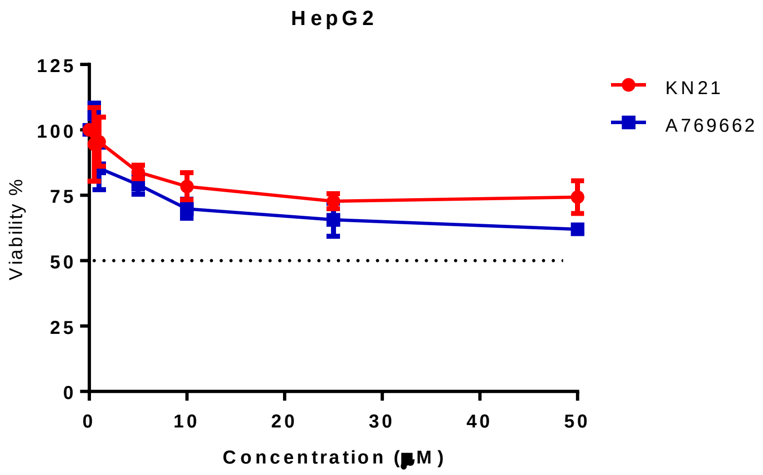

Compound (24 h)	Cytotoxicity (IC_{50}) HepG2
**KN21**	> 50
**A769662**	> 50

Figure S4. Cytotoxicity of KN21 and A769662 in HepG2 cells.

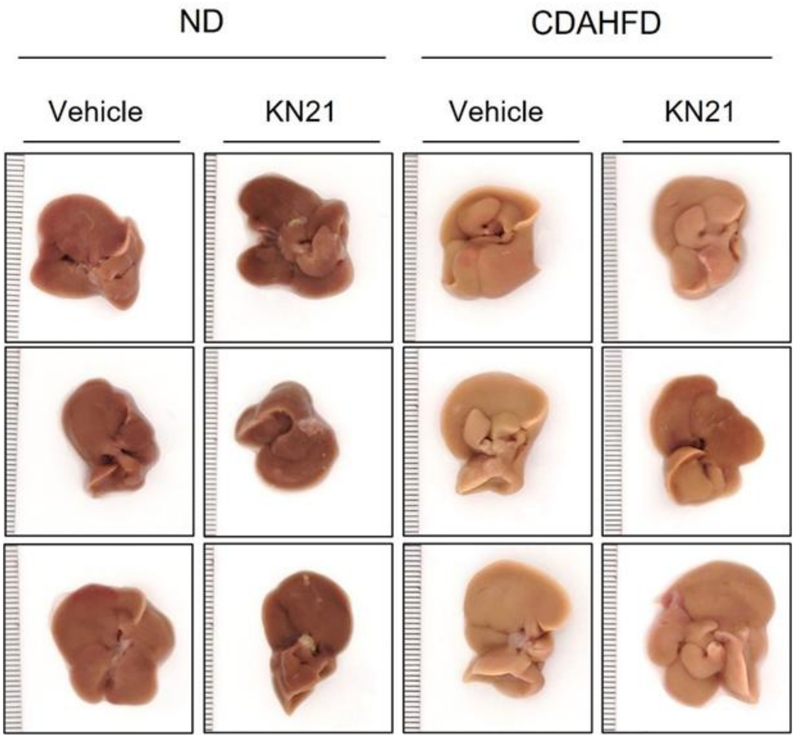

Figure S5. Additional liver images to complete the representation of all experimental groups in Figure 2 (n = 5).

Representative images of livers from mice in each experimental group (ND, CDAHFD, CDAHFD + KN21) to ensure a total of five liver samples per group, complementing Figure 2. These images further demonstrate the effects of KN21 treatment on liver morphology in the CDAHFD-induced mouse model of MASH.

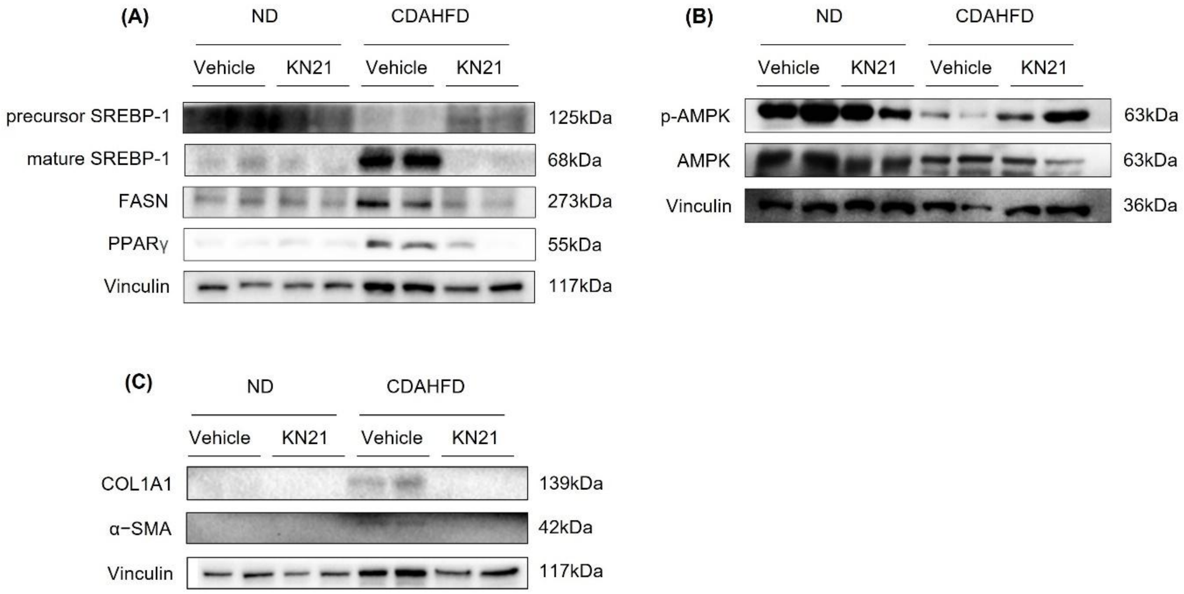

Figure S6. KN21 reduces lipogenesis (A) through AMPK activation (B) and mitigates liver fibrosis (C) in CDAHFD-fed mouse model.

The effects of KN21 on lipogenesis makers (SREBP-1, FASN and PPARγ), AMPK activity, and fibrotic markers (COL1A1 and α–SMA) were analyzed by western blotting in each group. These were performed to meet the condition (n=5) in Fig. 2J, 3A and 3D, respectively.

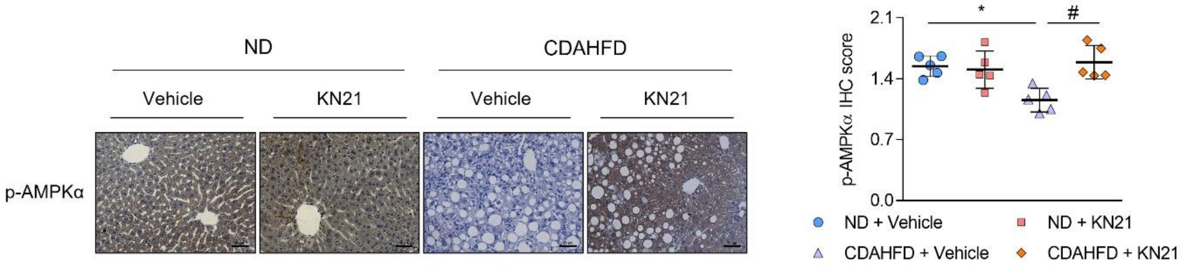

Figure S7. Immunohistochemical staining of phosphorylated AMPKα (p-AMPKα) in liver sections (n=5).

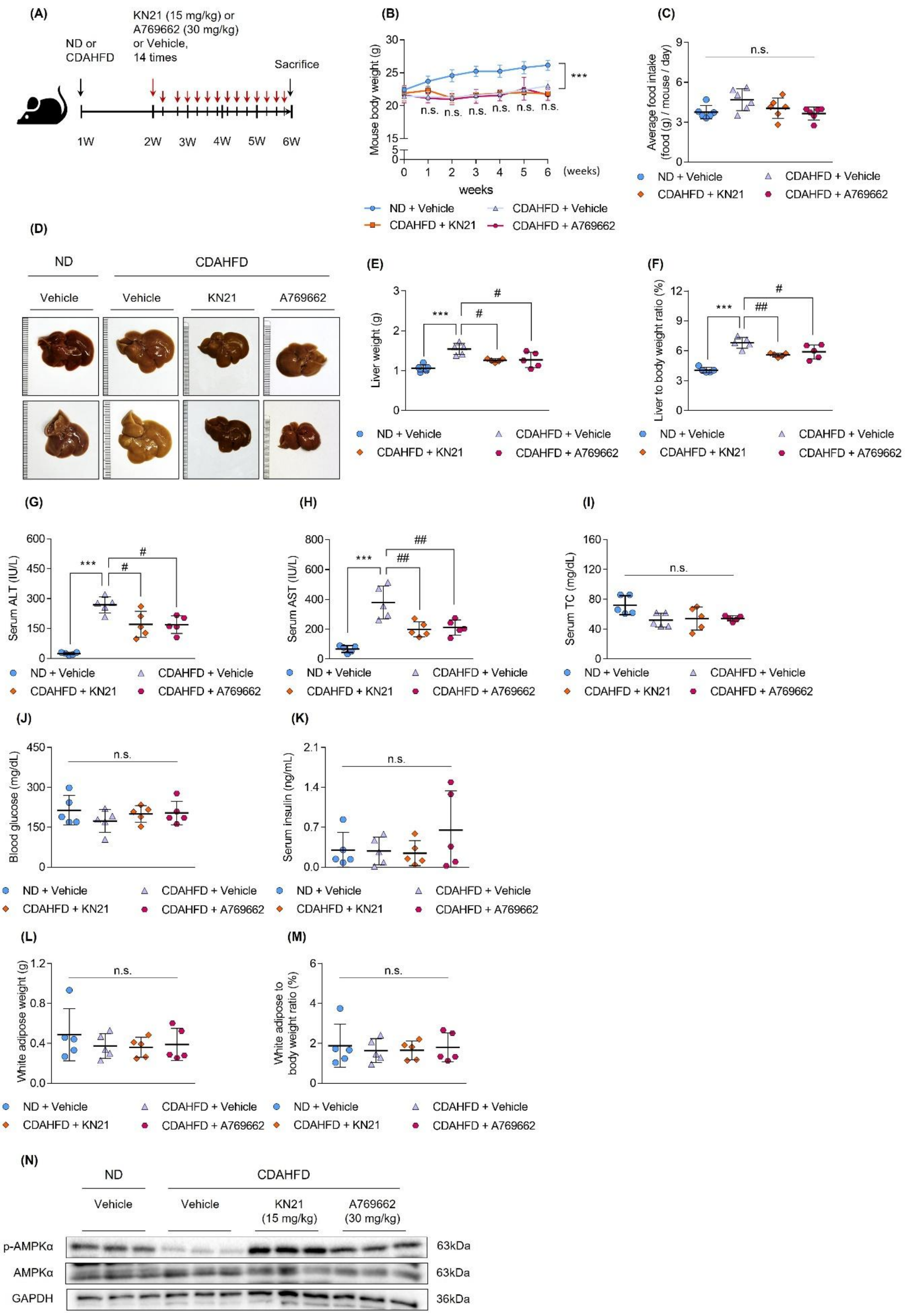
(A)
KN21 (15 mg/kg) or
A769662 (30 mg/kg)
or Vehicle,
14 times
ND or
CDAHFD
Sacrifice
1W
2W
3W
4W
5W
6W
(B)
Mouse body weight (g)
weeks
(weeks)
n.s.
***
ND + Vehicle
CDAHFD + Vehicle
CDAHFD + KN21
CDAHFD + A769662
(C)
Average food intake
(food (g) / mouse / day)
n.s.
(D)
ND
CDAHFD
Vehicle
Vehicle
KN21
A769662
(E)
Liver weight (g)
(F)
Liver to body weight ratio (%)
(G)
Serum ALT (IU/L)
(H)
Serum AST (IU/L)
(I)
Serum TC (mg/dL)
(J)
Blood glucose (mg/dL)
(K)
Serum insulin (ng/mL)
(L)
White adipose weight (g)
(M)
White adipose to
body weight ratio (%)
(N)
ND
CDAHFD
Vehicle
Vehicle
KN21
(15 mg/kg)
A769662
(30 mg/kg)
p-AMPKα
AMPKα
GAPDH
63kDa
63kDa
36kDa

Figure S8. KN21 and A769662 mitigate hepatic steatosis and liver damage in CDAHFD-fed mice via AMPK activation.

(A) Experimental timeline showing CDAHFD feeding and administration of KN21 (15 mg/kg) or A769662 (30 mg/kg). (B, C) Body weight and food intake of mice across treatment groups during the experimental period (n=5). (D) Representative liver images from each group. (E, F) Liver weights and liver-to-body weight ratio for each group (n=5). (G, H) Serum levels of ALT and AST as indicators of liver injury (n=5). (I, J, K) Serum levels of TC, glucose, and insulin. (L, M) Weights of subcutaneous and visceral white adipose tissue, and their ratio to total body weight (n=5). (N) Immunoblotting analysis of total and phosphorylated AMPKα in liver tissues of CDAHFD-fed mice treated with KN21 (15 mg/kg) or A769662 (30 mg/kg). $^{***}P < 0.001$, $^{**}P < 0.01$, $^{*}P < 0.05$ vs. the ND group; $^{\#\#\#}P < 0.001$, $^{\#\#}P < 0.01$, $^{\#}P < 0.05$ vs. the CDAHFD group (one-way ANOVA); "n.s." indicates a nonsignificant difference.

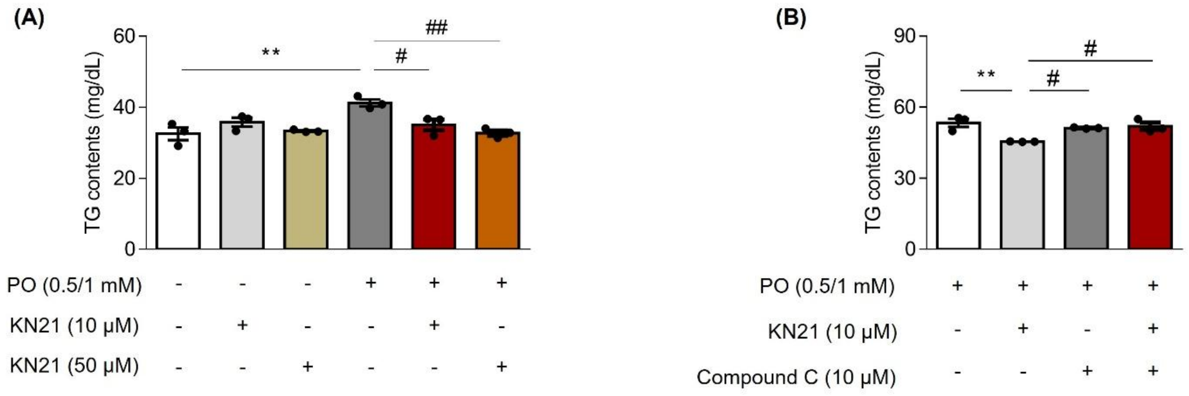

Figure S9. Effects of KN21 on lipid accumulation in primary hepatocytes.

(A) TG levels in primary hepatocytes stimulated with BSA or PO (0.5 mM PA and 1.0 mM OA) and treated with DMSO, 10 μM KN21, or 50 μM KN21 for 12 h. (B) TG levels of PO-stimulated primary hepatocytes treated with KN21, Compound C (AMPK inhibitor), or their combination for 12 h. Data indicate that KN21-mediated reduction in lipid accumulation is dependent on AMPK activation.

Table S1. Antibodies and others used in this study

Name	Supplier	Cat no.
Phospho-AMPKα (Thr172)	Cell signaling	2535
AMPKα1	Invitrogen	AHO1332
AMPKγ1	Cell signaling	4187
SREBP-1	Santa Cruz	Sc-365513
PPARr	Cell signaling	2435
FASN	Cell signaling	#3189
α-SMA	Genetex	GTX100034
Fibronectin	Genetex	GTX112794
Collagen 1A1	Genetex	GTX112731
α-tubulin	MBL	M175-3
Vinculin	MBL	PM088
GAPDH	MBL	M171-3
Goat Anti-rabbit secondary ab (HRP)	Genetex	GTX213110-01
Anti-mouse IgG ab (HRP)	Genetex	GTX213111-01
Recombinant Human TGF-β1	R&D systems	7754-BH-005
A769662	Tocris Bioscience	3336

Table S2. Primer Sequences used in this study

Name	Sequence	Supplier
mCOL1A1	Fw: GCTCCTCTTAGGGGCCACT<br>Rv: CCACGTCTCACCATTGGGG	BIONICS
mCOL3A1	Fw: CTGTAACATGGAAACTGGGGAAA<br>Rv: CCA TAGCTGAACTGAAAACCAC	BIONICS
mTIMP1	Fw: GCA ACT CGG ACC TGG TCA TAA<br>Rv: CGG CCC GTG ATG AGA AAC T	BIONICS
mPDGFB	Fw: CATCCGCTCCTTTGATGATCTT<br>Rv: GTGCTCGGGTCATGTTCAAGT	BIONICS
mPDGFA	Fw: GAGGAAGCCGAGATACCCC<br>Rv: TGCTGTGGATCTGACTTCGAG	BIONICS
mSREBF-1	Fw: TGACCCGGCTATTCCGTGA<br>Rv: CTGGGCTGAGCAATACAGTTC	BIONICS
mFASN	Fw: GGAGGTGGTGATAGCCGGTAT<br>Rv: TGGGTAATCCATAGAGCCCAG	BIONICS
mCD36	Fw: GACTGGGACCATTGGTGATGA<br>Rv: AAGGCCATCTCTACCATGCC	BIONICS
mGAPDH	Fw: AATGGTGAAGGTCGGTGTG<br>Rv: GTGGAGTCATACTGGAACATGTAG	BIONICS
hFASN	Fw: ACAGCGGGGAATGGGTACT<br>Rv: GACTGGTACAACGAGCGGAT	BIONICS
hPPARγ	Fw: TACTGTCGGTTTCAGAAATGCC<br>Rv: GTCAGCGGACTCTGGATTCAG	BIONICS
hSCD1	Fw: TCATAATTCCCGACGTGGCT<br>Rv: CCCAGAAATACCAGGGCACA	BIONICS
hCOL1A1	Fw: GGACACAGAGGTTTCAGTGG<br>Rv: CCAGTAGCACCATCATTTCC	BIONICS
hACTA2	Fw: AGTTACGAGTTGCCTGATGG<br>Rv: GAGGTCCTTCCTGATGTCAA	BIONICS
hFN	Fw: GTGTTGGGAATGGTCGTGGGGAATG<br>Rv: CCAATGCCACGGCCATAGCAGTAGC	BIONICS
hCTGF	Fw: CTTGCGAAGCTGACCTGGAA<br>Rv: GTGCAGCCAGAAAGCTCAAA	BIONICS
hGAPDH	Fw: CTTTGTCAAGCTCATTTCCTGG<br>Rv: TCTTCCTCTTGTGCTCTTGC	BIONICS
